# Influence of Fire Mosaics, Habitat Characteristics and Cattle Disturbance on Mammals in Fire-Prone Savanna Landscapes of the Northern Kimberley

**DOI:** 10.1371/journal.pone.0130721

**Published:** 2015-06-29

**Authors:** Ian J. Radford, Lesley A. Gibson, Ben Corey, Karin Carnes, Richard Fairman

**Affiliations:** 1 Department of Parks and Wildlife, Lot 248 Ivanhoe Rd (PO Box 942), Kununurra WA 6743, Australia; 2 Department of Parks and Wildlife, Locked Bag 104, Bentley Delivery Centre, WA 6983, Australia; Ecole Pratique des Hautes Etudes, FRANCE

## Abstract

Patch mosaic burning, in which fire is used to produce a mosaic of habitat patches representative of a range of fire histories (‘pyrodiversity’), has been widely advocated to promote greater biodiversity. However, the details of desired fire mosaics for prescribed burning programs are often unspecified. Threatened small to medium-sized mammals (35 g to 5.5 kg) in the fire-prone tropical savannas of Australia appear to be particularly fire-sensitive. Consequently, a clear understanding of which properties of fire mosaics are most instrumental in influencing savanna mammal populations is critical. Here we use mammal capture data, remotely sensed fire information (i.e. time since last fire, fire frequency, frequency of late dry season fires, diversity of post-fire ages in 3 km radius, and spatial extent of recently burnt, intermediate and long unburnt habitat) and structural habitat attributes (including an index of cattle disturbance) to examine which characteristics of fire mosaics most influence mammals in the north-west Kimberley. We used general linear models to examine the relationship between fire mosaic and habitat attributes on total mammal abundance and richness, and the abundance of the most commonly detected species. Strong negative associations of mammal abundance and richness with frequency of late dry season fires, the spatial extent of recently burnt habitat (post-fire age <1 year within 3 km radius) and level of cattle disturbance were observed. Shrub cover was positively related to both mammal abundance and richness, and availability of rock crevices, ground vegetation cover and spatial extent of ≥4 years unburnt habitat were all positively associated with at least some of the mammal species modelled. We found little support for diversity of post-fire age classes in the models. Our results indicate that both a high frequency of intense late dry season fires and extensive, recently burnt vegetation are likely to be detrimental to mammals in the north Kimberley. A managed fire mosaic that reduces large scale and intense fires, including the retention of ≥4 years unburnt patches, will clearly benefit savanna mammals. We also highlighted the importance of fire mosaics that retain sufficient shelter for mammals. Along with fire, it is clear that grazing by introduced herbivores also needs to be reduced so that habitat quality is maintained.

## Introduction

Patch mosaic burning (PMB), in which fire is used to produce a mosaic of habitat patches representative of a range of fire histories (‘pyrodiversity’), has been widely advocated to promote greater biodiversity [1], [2], [3], [4], [5]. The premise of PMB is that greatest biodiversity values will be maintained where habitat of varying post-fire intervals, fire frequencies and intensities is available to biota at an appropriate patch size. This is an extension of the habitat mosaic complexity hypothesis, that habitat complexity promotes biodiversity [6]. Parr and Andersen [5] critiqued the ‘pyrodiversity begets biodiversity’ paradigm, suggesting that not all fire patterns are ecologically meaningful, and emphasised the need for a more critical evaluation of the levels of pyrodiversity required for components of biodiversity to better inform management strategies. For example, Andersen et al. [7] reported that assemblage differences of tropical savanna ants were only detectable between long unburnt and frequently burnt areas, not subtle differences in fire patterns.

Small to medium-sized mammals (35 g to 5.5 kg) appear to be particularly sensitive to fire [8], [9]. This is at odds with many other savanna fauna which are resilient to many if not most fire regimes [10], [11], [12], [13], [14], [7]. Mammal populations in savanna landscapes have been shown to respond to single fire events [15], variations in fire season or intensity [11], [8], fire frequency [16], [9], and fire extent [17], [18]. However, which properties of fire mosaics are most instrumental in influencing mammal populations is still not clear. For example, some studies have demonstrated marked local population declines in areas of high fire intensity and extent e.g. [8], [15], while others show minimal impact, although this appears to be scale-specific [14]. This inconsistency and the fact that fire mosaic attributes are often correlated, makes it difficult to identify the relative importance of these in influencing mammal populations, and in many cases, responses are likely to be context and species specific. Additionally, some studies have been conducted in regions where populations have already suffered major declines e.g. [19], [9], [18]. Under these circumstances, low sample size makes it difficult to statistically describe patterns, and the level of replication necessary to detect patterns may be difficult to achieve given logistical and financial constraints [20]. It is also unclear what mechanisms underlie responses to fire [21], [22], [23], [14]. For example, within a given fire mosaic, mammals can respond to changes in food availability [24], [13], changes in vegetation structure and increased predator-prey interactions [25], [14], [26], [27], or to long term losses of key habitat attributes such as tree hollows, productivity and vegetation cover and complexity [25], [28], [29], [30].

Small and medium-sized mammals across much of Australia’s tropical savannas have suffered dramatic declines in the last few decades [31], [32], [25], [28], [9], [33], [34], [35], [30], [36]. These declines have been attributed to inappropriate fire regimes, resulting in broad scale reductions in fine-scale habitat complexity, combined with interacting factors including predation by the introduced feral cat *Felis catus* [33], [37], [26], [27] and degradation of habitats by introduced herbivores [38], [33]. One savanna region with a relatively intact mammal fauna is the Kimberley region of northern Western Australia [28], [39], [40], [41], [42], [30]. Although some declines are evident within parts of the region [31], [28], [39], including regional losses of larger (>150 g) marsupial and rodent species in lower rainfall zones [30], the high rainfall zone of the north-west Kimberley (>900 mm) still has areas with relatively high mammal abundance and species richness [42], [30], similar to those documented in the Northern Territory prior to recent mammal declines [32], [8], [9], [34]. Even so, similar to the other savanna regions in Australia, the fire regime of the north-west Kimberley has changed to a higher frequency of extensive and intense fires [43]. To redress this issue, a landscape-scale adaptive management program involving prescribed fire has been implemented in this region [42]. The program is aimed at reducing the overall area burnt each year, decreasing the proportion of the area burnt in the mid-late dry season (i.e. the time of year when fires are most intense), decreasing the size of burnt patches and increasing the proportion of vegetation in older age classes [42]. The program also aims to reduce cattle densities following findings that feral cattle have negative impacts on mammals [38]. Monitoring changes in the richness and abundance of small to medium-sized mammals is a further component of this program.

In the current study, we utilise the monitoring data above, and remotely sensed fire scar imagery, to examine the influence of attributes characterising fire mosaics (i.e. time since fire, fire frequency, frequency of late dry season fires, diversity of post-fire ages in 3 km radius, and spatial extent of recently burnt, intermediate and long unburnt habitat) on the abundance and richness of mammal species in the north Kimberley. Additionally, as variables describing the habitat at each site were also available (along with an index of cattle disturbance), we included those in our assessment. It was of added interest to investigate the importance of habitat attributes representing shelter availability for small and medium-sized mammals. To avoid unexplained variation between years, we only include data recorded during 2013, the year when most sites were sampled and with the same sampling effort. We aimed to improve our understanding of the relative importance of fire mosaic characteristics, and available shelter, for small to medium-sized mammals in the savanna landscapes of this region to inform fire management practices.

## Methods

### Study area

**Fig 1:**
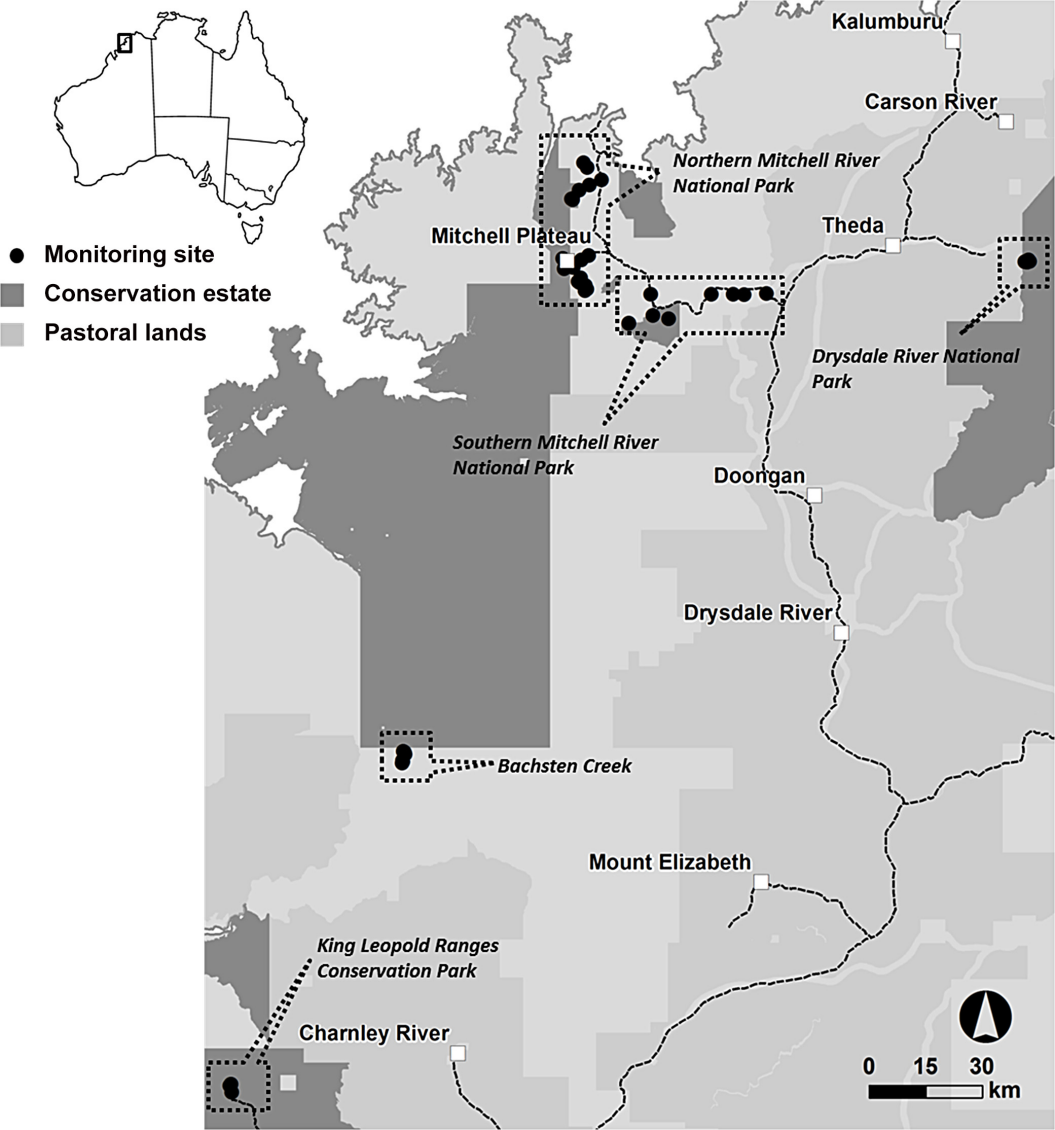
Locations of survey sites in the north Kimberley region of northern Western Australia.

The study area is located in the North Kimberley biogeographic region of north-western Western Australia (Fig 1). The region experiences a tropical monsoonal climate, with high temperatures year round (daily mean maximum 29.6–36.0°C), and high rainfall (1550 mm to 900 mm annual) occurring predominantly during the warmer months from November to April. Savanna vegetation is characteristic of this region with a range of eucalypt species making up the tree canopy in most areas and grasses dominating the understory with usually a relatively sparse shrub layer. Vegetation ranges from savanna forest (30–50% tree cover) through to woodland (10–30% cover) and shrubland (< 10% cover) depending on substrates, and grass cover ranges from 5 to almost 100% projected ground cover. Small patches of rainforest and riparian forest with sometimes closed forest canopies (> 70% cover) and zero grass cover occur within the savanna matrix. Substrates range from relatively fertile clay soil on igneous rock, through laterite derived loam and gravel substrates, to sandy or skeletal soils, or rugged sandstone outcrops. Due to the annual cycle of a wet season, followed by an extended dry season (>6 months) in which grasses cure, savannas in the region are subject to regular fires [44]. The North Kimberley region has not been subject to extensive clearing but fire regimes have putatively changed to more frequent and intense fires in recent decades [43]. Recent changes in fire regimes are often linked with the breakdown of traditional indigenous fire management in many areas across northern Australia [44]. Introduced herds of cattle, horses and donkeys are present throughout the region and grazing regimes have also intensified since pastoralism was introduced in the 1920s [38], [43], [26].

### Survey design

Study sites were distributed between five sub-regions within the North Kimberley: the Northern Mitchell River National Park, Southern Mitchell Plateau, Drysdale River National Park, King Leopold Range National Park and Bachsten Creek at the southern edge of Prince Regent National Park (Fig 1). Study sites were stratified between habitat types in each subregion. Habitat types were based on geology (volcanic, laterite and sandstone) and habitat/vegetation structure (woodland, forest and rock scree). Location of sites was based on habitat stratification, and where possible, the location of historical survey sites e.g. [45], [39], [14], [42], [30]. Sites were located both inside and outside National Parks to account for possible disturbance factors within the region, and were deliberately distributed as widely as possible given major limitations to vehicle access. Here, we analyse the data compiled for 49 sites sampled in 2013 – the maximum number of sites sampled in any one year and with consistent sampling effort across all sites.

### Mammal data

Mammal data were collected from 50 by 50 m quadrat arrays similar to those used as a standard monitoring plot in other areas of northern Australia [32], [20], [9]. A total of 20 metal box traps (Elliotts), alternately large (15 x 15.5 x 46 cm) and medium (9 x 10 x 33 cm), were placed around the perimeter of each quadrat. Four larger wire cage traps (25 x 30 x 73 cm) were placed at the corners. Traps were baited with a mixture of peanut butter and rolled oats. Traps were shaded using grass, leaves or hessian sacks to prevent overheating. They were placed at sites for five nights making a total of 120 trap nights for each site. Traps were checked early each morning to prevent overheating. Mammals were identified to species and marked using microchips for larger species or permanent marker pen on the ears for small rodents. Site mammal data were described in terms of abundance (total number of individuals captured not including recaptures) and species richness (total number of species captured).

### Habitat attributes

Each study site was assessed for habitat attributes known to be affected by fire [10], [12], [16] and large herbivores [46] and/or which may influence mammal assemblages [47], [21], [14] (Table 1). Tree (height > 4 m) and shrub (height < 4 m) projected canopy cover were estimated using a 1% Bitterlich gauge [48]. Assessing each canopy in a 360° arc from the central point gives a total percentage value for canopy cover. Ground vegetation cover was assessed using a 50 m transect run diagonally from the corner post. The total distance under the transect tape of cover of perennial grass (tussock or hummock), annual grass (*Sorghum* spp.), forbs and subshrubs (<50 cm high) was added to calculate total percentage cover value for ground layer vegetation. Leaf litter cover and exposed rock or gravel cover were measured using the same transect based method. Indices of rock crevice, tree hollows and fallen timber availability were estimated as: none (0), some (1), low (2), moderate (3), high (4) or extensive (5). Values for latter two variables were added together to give a single index. Cattle disturbance was qualified as cattle sighted [none (0), single individual (1), several (2), groups (3)], grazing level and evidence of tracks/trampling [no evidence (0), light (1), moderate (2), heavy (3)] and cattle dung [none (0), some sighted (1), scattered (2), extensive (3)]. These values were added to give an index of cattle disturbance. While arbitrary these indices describe the range of variation seen among sites in the field.

**Table 1:** Explanatory variables measured at survey sites, their definitions and summary statistics. Variables in bold were included in the final set for analyses.

Name	Abbrev.	Description	Range	Mean
*Habitat Attributes*				
Tree canopy cover (%)	**Tcov**	Projected tree canopy cover as a percentage	6–70	22.4
Rock cover (%)	**Rcov**	Percentage ground cover of rocks and gravel	0–76	22.5
Shrub canopy cover (%)	**ShCov**	Projected shrub canopy cover as a percentage	0–85	11.0
Litter cover (%)	**Litt**	Percentage of ground covered by leaf and woody litter	2–97	28.5
Vegetation ground cover (%)	**VegGr**	Percentage of ground covered by grasses, forbs and sub-shrubs (<50 cm height)	0–99	42.0
Rock crevices (index)	**Rcrev**	Index of abundance of rock crevices available as fauna habitat	0–5	2.1
Tree hollows and logs (index)	**HoLo**	Index of abundance of trees with apparent hollows and logs available as fauna habitat	2–10	4.4
Cattle index	**CattIn**	Index of cattle disturbance including those sighted, trampling, dung and grazing impacts	0–11	2.7
*Fire mosaic attributes*				
Fire frequency	**FF**	Number of times site burnt from 2003 to 2012	3–9	6.8
Time since last burnt (years)	**TSLB**	The time from site survey to the most recent fire at the site	0–4	0.6
Frequency late dry season fire (10 years^-1^)	**FF_LDS**	Frequency of fires in the last 10 years which occurred from July to December	0–8	4.1
Average distance to edge most recent fire (m)	medge	Mean linear distance of site to the nearest unburnt vegetation in previous year (correlated with X1yr, *r* = 0.76)	0–2225	509
Average distance to edge over 10 yr (m)	**aedge**	Mean linear distance of site to the nearest unburnt vegetation for fires in the last 10 years	33–2443	1077
Diversity of post-fire age	Divers	Number of categories of TSLB within 3 km radius of site with >1% coverage (correlated with LU4yr, *r* = 0.89)	2–8	3.3
Extent of recent fire 1 yr old (%)	**X1yr**	Percentage of 3 km radial area around site burnt in previous year (last burnt in 2012)	0–92	37.5
Extent medium fire 2–3 yr old (%)	**X23yr**	Percentage of 3 km radial area around site burnt in 2 to 3 years prior to survey (last burnt in 2010–2011)	0–31	7.7
Extent ≥4 yr old (%)	**LU4yr**	Percentage of 3 km radial area around site burnt in ≥4 years prior to survey (last burnt 2009 or before)	0–16	2.0

### Fire mosaic data

Remotely sensed data was used to derive fire mosaic attributes (Table 1). Analyses are based on fire scars derived from Moderate Resolution Imaging Spectroradiometer (MODIS) imagery at 250 m resolution (http://modis.gsfc.nasa.gov/). MODIS data for each year were obtained from the North Australia Fire Information (NAFI) website (http://www.firenorth.org.au/nafi2/). Time since last fire (TSLB), fire frequency (FF) and frequency of late dry season fires over the previous 10 years (2003 to 2012 –FF_LDS) was calculated for each site surveyed in 2013. As an indication of fire size or extent, a raster analysis was used to determine the mean distance from each survey site to the nearest unburnt vegetation patch greater than 20 ha in size (three MODIS pixels). Distance from each site to the nearest unburnt edge in the previous year was calculated and a mean value was also calculated for the distance to unburnt vegetation for all years where the site was burnt in the years from 2003 to 2012. Further calculations were made for fire mosaics surrounding study sites based on the area inside concentric rings within 1 km, 3 km and 5 km radii from each site. Within each radial area, area of habitat (expressed as a proportion) the number of post-fire age classes where greater than one per cent was burnt, was used to represent fire mosaic diversity (1 to 13 post-fire ages). These multiple post-fire ages were categorised into three classes for classification of generalised fire regime in surrounding areas: 1 year since the last fire, 2 to 3 years since fire and ≥4 years unburnt. All spatial analyses were carried out in ArcMap 10.1 using tools in the Spatial Analyst extension.

### Data analysis

The *caret* package in the R statistical computing language R Development Core Team [49] was used to reveal inter-correlations among candidate variables (i.e. >0.7), and to provide a criterion for reducing the variable set and thereby avoid over-fitting in the subsequent modelling. The extent of area burnt categories within 1 km, 3 km and 5 km radii of survey sites were strongly inter-correlated and only the 3 km radius data were retained for analysis. This distance was considered appropriate as it encompasses the known home range sizes of all the species detected [50], [51], [52], [53], [54], [55], [56], [57] and because preliminary univariate analyses showed stronger correlations between mammal and fire attributes at the 3 km radius. Among fire mosaic attributes, mean distance from site location to unburnt edge during the previous 10 and number of post-fire age categories within a 3 km radius of each study site were also removed from further analyses due to inter-correlations (Table 1). Cattle disturbance indices were pooled prior to analysis.

Fourteen variables were available for analysis, once correlated variables were removed, and we used a staged process (see Williams et al. [58]) to reduce the variable-set even further. In the first step, the 14 variables were separated into two subgroups: habitat-specific attributes (including the cattle disturbance index) and fire-specific attributes. The association between mammal abundance (both total abundance and for selected individual species with sufficient sample size), and species richness, and all possible subsets of the variables within each of these subgroups were modelled using a Generalised Linear Model (GLM). To correct for overdispersion in the abundance data, we used a negative binomial distribution; otherwise a Poisson distribution was considered appropriate. Zero-inflated regression models were also examined for the single-species models, but as these tended to perform no better or worse, we only present the results for negative binomial GLMs. The use of GLMs was considered appropriate based on predominantly linear response curves during inspections of mammal and fire/habitat variable responses using Generalised Additive Models. Model ranking from best to worse was based on the second-order Akaike Information Criterion (AICc) [59]. Only the variables included in the top-ranked model for both subgroups were included in a final candidate set. We included all models in the final candidate sets for model averaging, where inference is based on a set of plausible models to estimate parameters. We also calculated AICc weights (*wi*) and used these to weight model coefficients. The relative importance of covariates was examined by summing the AICc weights for each covariate across all models in which it occurred in (w+; [59]). Data analyses were run in the R statistical computing language [49] and the contributed *MuMIn* and *MASS* packages. We evaluated the fit of the averaged-model by regressing the fitted values against observed values.

## Results

### Mammal fauna

In 2013, 375 individual mammals were captured during 49 site surveys and 5880 trap nights, with a range of 0 to 25 animals captured per site (mean of 7.6) (Table 2). The most highly detected mammal group was the medium and small rodents (<150 g) with a total of 250 individuals recorded at 36 sites (Table 2). Of these, the most commonly captured were *Zyzomys argurus* and *Pseudomys nanus* and models were produced for these two species (Table 2). *Dasyurus hallucatus*, a carnivorous marsupial, was the next most highly detected species with 62 individuals captured at 13 sites (mean abundance 1.27 per site) and the abundance of this species was also modelled. Two bandicoot species, *Isoodon auratus* and *I*. *macrourus*, were detected at 6 and 9 survey sites respectively, with a mean combined abundance of 0.56 per site (Table 2). The larger arboreal rodents, *Mesembriomys macrurus* and *Conilurus penicillatus*, were detected at 7 sites with a combined mean abundance of 0.39 per site (Table 2). Few small dasyurids (*Sminthopsis virginiae* and *Pseudantechinus ningbing*), possums (*Wyulda squamicaudata*) or small macropods (*Petrogale burbidgei*) were captured during the surveys (Table 2). Overall mean species richness was 1.96 per site with a range in richness of 0–7 species per site (Table 2).

**Table 2:** Mammal species captured at 49 sites during 2013.

Species	Common name	Total abundance	Range	Mean	No. present
**Medium & small rodents**					
*Zyzomys argurus*	Common rock-rat	84	0–14	1.71	16
*Pseudomys nanus*	Western chestnut mouse	55	0–19	1.12	14
*Melomys burtoni*	Grassland melomys	46	0–18	0.94	7
*Rattus tunneyi*	Pale field rat	31	0–10	0.63	7
*Zyzomys woodwardi*	Kimberley rock-rat	26	0–5	0.53	8
*Pseudomys delicatulus*	Delicate mouse	6	0–2	0.12	4
*Pseudomys johnsoni*	Pebble mound mouse	2	0–1	0.04	2
**Carnivorous marsupials**					
*Dasyurus hallucatus*	Northern quoll	62	0–13	1.27	13
**Bandicoots**					
*Isoodon auratus*	Golden bandicoot	13	0–5	0.27	6
*Isoodon macrourus*	Northern brown bandicoot	14	0–3	0.29	9
**Arboreal rodents**					
*Mesembriomys macrurus*	Golden-backed tree-rat	12	0–4	0.25	7
*Conilurus penicillatus*	Brush-tailed rabbit-rat	7	0–5	0.14	3
**Small dasyurids**					
*Sminthopsis virginae*	Red-cheeked dunnart	8	0–5	0.14	4
*Pseudantechinus ningbing*	Ningbing false antechinus	2	0–1	0.04	2
**Macropods**					
*Petrogale burbidgei*	Monjon	6	0–3	0.12	4
**Possums**					
*Wyulda squamicaudata*	Scaly-tailed possum	1	0–1	0.02	1
**Mammals**					
Total		375	0–25	7.61	41
Richness		16	0–7	2.16	

Species common name, total number captured, range of captures per site, mean captures per site (from 120 trap nights) and number of sites where each species was recorded.

### Total mammal abundance and richness

Mammal abundance was strongly influenced by a negative association with frequency of late dry season fires (FF_LDS), extent of area burnt less than 1 year prior to survey (X1yr), and tree canopy cover (Tcov), and a positive association with shrub canopy cover (ShCov) (Table 3). Mammal abundance also tended to decline with increasing cattle disturbance (CattIn) (Table 3). The most-supported model included all these attributes and explained 40% of the deviance in mammal abundance among study sites (Table 4). The averaged-model performed moderately well (Adjusted R-square = 0.47, P <<0.001). Similar results were observed for mammal richness, but there was only weak support for the cattle disturbance and shrub cover variables, and neither were retained in the top-ranked model (Tables 3 & 4). In this case, the most supported model explained 24.9% of the deviance in species richness among sites (Table 4), and the model fit of the averaged-model was moderate (Adjusted R-square = 0.31, P<<0.001).

**Table 3:** Model-averaged coefficients and standard errors for each variable included in the modelling of abundance and richness of mammals at monitoring sites in the North Kimberley.

Variable	Coefficient	Standard Error	*W*+
**Mammal abundance**			
Intercept	4.502	0.688	-
FF_LDS	-0.420	0.109	0.99
X1yr	-0.013	0.005	0.92
Tcov	-0.029	0.012	0.87
ShCov	0.023	0.010	0.71
CattIn	-0.087	0.048	0.62
**Species richness**			
Intercept	2.038	0.535	-
X1yr	-0.010	0.004	0.88
FF_LDS	-0.198	0.078	0.87
Tcov	-0.015	0.008	0.63
CattIn	-0.072	0.045	0.55
ShCov	0.007	0.008	0.32
***Dasyurus hallucatus***			
Intercept	3.092	2.342	-
Tcov	-0.097	0.041	0.84
CattIn	-0.342	0.164	0.70
X1yr	-0.020	0.013	0.50
aedge	-0.001	0.001	0.43
Rcrev	0.313	0.291	0.35
FF	-0.323	0.299	0.33
***Pseudomys nanus***			
Intercept	-6.180	3.234	-
VegGr	0.032	0.016	0.77
aedge	0.002	0.001	0.75
LU4yr	0.284	0.148	0.72
HoLo	0.303	0.195	0.57
FF	0.563	0.413	0.46
***Zyzomys argurus***			
Intercept	-0.578	1.370	-
Rcrev	0.816	0.239	1.00
CattIn	-0.592	0.240	0.96
Tcov	-0.065	0.027	0.93
FF	-0.012	0.183	0.22

Sum of weights (i.e. relative importance) for models containing each coefficient *w*+ are also shown.

**Table 4:** Results of AIC*c*-based model selection for total mammal abundance, mammal species richness, and abundance of *Dasyurus hallucatus*, *Pseudomys nanus* and *Zyzomys argurus* at monitoring sites (n = 49) in the North Kimberley.

Models	*K*	logLik	AICc	Δ*i*	*wi*	%Dev
**Mammal abundance**						
Tcov+ShCov+CattIn+FF_LDS+X1yr	7	-137.95	292.63	0.00	0.37	40.00
Tcov+ShCov+FF_LDS+X1yr	6	-139.65	293.30	0.67	0.26	36.00
**Mammal richness**						
Tcov+FF_LDS+X1yr	4	-83.27	175.44	0	0.19	24.92
Tcov+CattIn+FF_LDS+X1yr	5	-82.19	175.78	0.34	0.16	27.86
FF_LDS+X1yr	3	-84.95	176.44	0.99	0.12	20.33
CattIn+FF_LDS+X1yr	4	-83.84	176.58	1.14	0.11	23.36
Tcov+ShCov+FF_LDS+X1yr	5	-82.74	176.87	1.42	0.09	26.38
Tcov+ShCov+CattIn+FF_LDS+X1yr	6	-81.56	177.12	1.68	0.08	29.58
***Dasyurus hallucatus***						
CattIn+Tcov	4	-55.37	119.66	0.00	0.12	37.65
Rcrev+Tcov+CattIn+aedge+X1yr	6	-53.18	120.36	0.70	0.08	46.40
Tcov+CattIn+X1yr	5	-54.60	120.60	0.95	0.07	40.66
Tcov+CattIn+aedge	5	-54.89	121.18	1.52	0.06	39.87
Tcov+CattIn+FF	5	-55.07	121.54	1.89	0.05	39.17
***Pseudomys nanus***						
VegGr+LU4yr+HoLo+aedge	6	-50.2	114.4	0	0.16	47.67
LU4yr+FF+aedge	5	-51.68	114.76	0.36	0.13	41.67
VegGr+LU4yr+FF+aedge	6	-50.41	114.81	0.41	0.13	46.67
VegGr+HoLo	4	-53.14	115.2	0.8	0.11	35.85
VegGr+HoLo+aedge	5	-52.23	115.86	1.46	0.08	39.64
VegGr+LU4yr+aedge	5	-52.3	116	1.6	0.07	38.86
VegGr+LU4yr+HoLo+FF+aedge	7	-49.64	116	1.6	0.07	49.74
***Zyzomys argurus***						
Rcrev+Tcov+CattIn+FF	5	-49.66	110.71	0	0.7	77.88

Only the most supported models with AICc differences <2 are presented. Number of model parameters (*K*), maximised log-likelihood values (logLik), AIC*c* values (AIC*c*), AIC*c* differences (Δ*i*), Akaike weights () and percent of deviance explained (%Dev) are shown for each of the models. See Table 1 for abbreviations of habitat and fire attributes.

### Dasyurus hallucatus

A negative association of the abundance of *Dasyurus hallucatus* with tree canopy cover and cattle disturbance was strongly supported (Table 3). Moderate to weak support for negative associations with extent of area burnt less than one year prior to survey, distance to the nearest unburnt patch (aedge) and fire frequency (FF), and a positive association with abundance of rock crevices was also observed (Table 3). The top-ranked model which explained 37.7% of the deviance only included the tree cover and cattle disturbance variables (Table 4). Both of these variables were also retained in all of the five most supported models (Table 4). The performance of the averaged-model was moderate (Adjusted R-square = 0.49, P<<0.001).

### Pseudomys nanus

Abundance of *Pseudomys nanus* was strongly related to a positive association with ground vegetation cover (VegGr), distance to the nearest unburnt patch (aedge) and extent of area burnt ≥4 years ago (LU4yr) (Table 3). Weaker (positive) associations with abundance of tree hollows and logs (HoLo), and fire frequency were also apparent (Table 3). The top-ranked model explained 47.7% of the deviance (Table 4). Model fit of the averaged-model was moderate (Adjusted R-square = 0.48, P<<0.001).

### Zyzomys argurus

*Zyzomys argurus* abundance was strongly associated with increasing abundance of rock crevices and decreasing cattle disturbance, tree canopy cover and fire frequency, although the latter variable was weakly supported and was not retained in the top-ranked model (Tables 3 & 4). The top-ranked model was the only model with substantial support, explaining 77.9% of the deviance (Table 4). The performance of the averaged-model was relatively high (Adjusted R-square = 0.51, P<<0.001).

## Discussion

### Influence of fire mosaic attributes

Fire mosaic attributes with the greatest support in terms of influencing mammal abundance and richness in the North Kimberley study area were frequency of late dry season fires and the extent of recently burnt habitat (post-fire age <1 year) within the surrounding 3 km of study sites. Abundance and richness of mammals tended to be higher in areas where there were fewer late dry season fires. While negative associations with fire frequency were observed for two of the individual species modelled (*Dasyurus hallucatus* and *Zyzomys argurus*), this influence was relatively weak, and the relationship was actually positive for *Pseudomys nanus* abundance (though again weak). These results suggest that fire frequency on its own is not highly influential, but that seasonality of fires is important. In a review of fire management for fauna conservation in Australian tropical savannas, Andersen et al. [8] likewise state that high intensity late dry season fires have major impacts on small mammals. Legge et al. [15] measured short-term responses of mammals to a large-scale late dry season fire in the central Kimberley, and found that richness and abundance was significantly lower in burnt compared to adjacent unburnt areas. Due to the build-up of dry biomass, fires at the end of the dry season tend to be more intense [60]. Such intense fires disproportionately alter both the habitat used by mammals, such as ground vegetation and shrub cover and the presence of large hollow-bearing trees [50], [12], [10], [13], as well as the connectivity of suitable habitat within the landscape [17].

The negative association of mammal abundance and richness with the extent of recently burnt habitat observed here is consistent with another recent study by Lawes et al.[18]. It appears that the spatial extent of post-fire habitat is as important as site specific fire history attributes in determining mammal assemblage status. Extensive recently burnt vegetation in an area is likely to result in fewer available refuges from fire (i.e. both unburnt habitat and habitat with sufficient vegetation cover), and therefore fewer sources of recolonisation [3], [17]. Large-scale fires also reduce dispersal opportunities into unburnt patches. Of the three individual species considered, the negative association of this variable on abundance was also observed for *D*. *hallucatus*.

The extent of ≥4 years unburnt habitat was positively related to *P*. *nanus* abundance. This result is consistent with this species’ strong positive association with ground vegetation cover also observed here. Apart from *P*. *nanus*, the local extent of ≥4 years unburnt habitat was not strongly supported in the models. As this variable was highly correlated (Pearson correlation = 0.9) with the number of post-fire age classes in the local area (i.e. pyrodiversity), a similar result can be inferred. Thus we also found little support for pyrodiversity promoting mammal richness or abundance. It is possible that the richness of mammal species recorded (maximum of 7) was too low to detect an effect, compared to other studies examining responses of ant communities (maximum richness of 162) where a diversity of fire ages promoted species diversity e.g. Maravalhas and Vasconcelos 2014 [61]. However, Kelly et al. [62] also found no relationship between small mammal richness and diversity of fire-ages in a fire-prone mallee ecosystem of semi-arid Australia. Similarly, Pastro et al.[63] reported no response in the diversity of arid-zone small mammals to small patchy prescribed burns, but a decline in diversity in response to a large-scale wildfire. Perhaps many small-medium sized mammal species are likely to occur in a wide range of post-fire age classes, and for fire-sensitive species, habitat suitability depends on the extent of recently burnt/unburnt habitat within their home range, rather than a mix of fire ages.

### Influence of habitat attributes and cattle disturbance

At the site-level, the importance of habitat attributes that constitute shelter for mammals was evident in the current study. Shrub cover was positively related to mammal abundance and richness, and availability of rock crevices, ground vegetation cover and presence of hollow logs and tree hollows were all positively associated with at least some of the mammal species modelled. Conversely, projected tree canopy cover was negatively related to mammal abundance and species richness, and both *D*. *hallucatus* and *Z*. *argurus* abundance. This result reflects the influence of geology (volcanic, laterite or sandstone) and vegetation structure (woodland, forest or rock scree) on mammal patterns (see Appendix 1). Here, mean abundance and richness of mammals was considerably lower in laterite woodland and rainforest sites where tree canopy cover was highest; compared to sandstone substrates (woodland and scree) and volcanic woodland where relatively high abundance and richness coincided with lower tree canopy cover. Similarly, *D*. *hallucatus* and *Z*. *argurus*, two species known to be associated with rocky habitats [45], [41], were almost exclusively recorded in either sandstone scree or sandstone woodland where tree cover was lower.

**Appendix 1:** Mean values of mammal abundance (including three species modelled) and richness, and explanatory variables (see Table 1 for definitions) for sites sampled within each habitat type (No. sites = number of sites sampled).

	Laterite woodland	Rainforest	Sandstone scree	Sandstone woodland	Volcanic woodland
Abundance					
Total	3.0	8.8	10.1	9.8	6.2
*Dasyurus hallucatus*	0.0	0.0	2.3	3.1	0.1
*Zyzomys argurus*	0.0	0.4	4.8	2.8	0.0
*Pseudomys nanus*	1.0	0.4	0.0	0.2	2.9
Species richness	1.4	2.0	2.7	2.8	1.9
Tcov	34.0	52.2	15.6	18.8	14.6
Rcov	18.6	26.2	46.6	22.2	7.2
ShCov	7.3	30.0	10.2	8.8	8.7
Litt	42.3	68.5	18.2	25.6	17.9
VegGr	38.0	13.2	31.2	43.4	59.7
Rcrev	1.6	2.8	4.1	2.7	0.3
HoLo	7.4	5.0	4.1	3.9	3.5
CattIn	0.7	4.2	1.2	1.2	5.3
FF	7.0	7.0	6.1	6.5	7.4
TSLB	0.7	0.8	0.8	0.8	0.3
FF_LDS	4.6	3.2	3.9	4.3	4.2
aedge	1149.4	980.4	981.4	1093.9	1126.5
X1yr	31.6	45.2	32.5	20.8	54.3
X23yr	12.4	4.2	8.5	9.4	4.9
LU4yr	1.6	2.0	2.7	2.1	1.6
No. sites	7	5	10	12	15

The negative effect of cattle disturbance was evident for total mammal abundance and species richness, and the abundance of both *D*. *hallucatus* and *Zyzomys argurus*. Few studies have specifically addressed the impacts of grazing on fauna in the tropical savannas of Australia, but one study in the central Kimberley showed that the abundance and richness of small mammals increased in response to destocking of introduced herbivores (cattle, donkeys and horses) [38]. Grazing by introduced herbivores is likely to interact with intense and large scale fires to simplify the vegetation structure [64]. Not only does this interaction reduce the habitat quality for small mammals by reducing vegetation cover, but also may facilitate increased predation of small mammals [21], [14], including by feral predators such as cats, by improving access [26], [27]. A recent study by McGregor et al. [26] showed that rates of hunting activity by feral cats was higher in recently and intensely burnt and heavily grazed habitats relative to unburnt and less-grazed areas. This study also showed that cats had a preference for open habitats with higher numbers of small mammals. Therefore increased cat activity under a regime of intense fires and high grazing pressure, is likely to increase predation rates on small mammals [26].

## Conclusions

Our results indicate that both a high frequency of intense late dry season fires and extensive, recently burnt vegetation are likely to be detrimental to savanna mammals in the North Kimberley. While we found no correlation between mammal diversity and diversity of post-fire age classes, a fire mosaic that reduces large scale and intense fires, including the retention of ≥4 years unburnt vegetation (long unburnt in the savanna context), will clearly benefit small mammals in these landscapes. Along with fire, it is clear that grazing by large herbivores (cattle, donkeys and horses) also needs to be carefully managed so that habitat quality is maintained. The importance of fire mosaics that retain sufficient shelter for mammals (e.g. shrub and ground cover, and hollow bearing trees and logs) was also highlighted. Continued monitoring in the North Kimberley will provide site data from multiple years. This extended dataset will allow an examination of whether patterns in mammal abundance and richness in response to managed fire mosaics are consistent across years, and thereby provide further insight into the key properties of fire mosaics that enhance the conservation of savanna mammals in the North Kimberley.
